# EHMTI-0040. Headaches after traumatic spinal cord injury

**DOI:** 10.1186/1129-2377-15-S1-B30

**Published:** 2014-09-18

**Authors:** L Sabre, M Rugo, J Kõrv, M Braschinsky

**Affiliations:** 1 Depratment of Neurology and Neurosurgery, University of Tartu, Tartu, Estonia; 2 Faculty of Medicine, University of Tartu, Tartu, Estonia

## Background

Patients with traumatic spinal cord injury (TSCI) often suffer from different types of pain. However, headaches (HA) after TSCI have not been studied specifically.

## Aim

To examine the HAs among patients with TSCI.

## Methods

The cross-sectional study included individuals with TSCI from 1997 to 2011 who were interviewed via telephone. The interview based on a specifically designed questionnaire.

## Results

There were 9 women and 64 men (mean age 37.1 ± 10.6 years). The most frequently mentioned pain was HA (71%), followed by back pain (60%) and pain in neck (44%). HAs were more frequent after the trauma compared with the HAs before TSCI (p=0.01). The HAs that arose after TSCI were not related to the concomitant brain injury (p=0.80). The occurrence of HA did not depend on the severity nor the level of the TSCI.

The most frequently reported HA located in the occipital, was pulsating and lasted from 1 to 3 hours. The maximal intensity of the pain was 6.9 ± 2.0 according to the Numeric Rating Scale.

Due to the HA 85% of the patients were not seen by any physician and their HA was not diagnosed.

## Conclusions

This is the first study that shows that HA is the most prevalent pain after TSCI. Despite this, the majority of patients are never consulted, diagnosed or appropriately managed due to their HA. This indicates that further studies are needed to provide evidence regarding the causes of HA and their impact on quality of life.

No conflict of interest.

